# Male patients affected by mosaic *PCDH19* mutations: five new cases

**DOI:** 10.1007/s10048-017-0517-5

**Published:** 2017-07-01

**Authors:** I. M. de Lange, P. Rump, R. F. Neuteboom, P. B. Augustijn, K. Hodges, A. I. Kistemaker, O. F. Brouwer, G. M. S. Mancini, H. A. Newman, Y. J. Vos, K. L. Helbig, C. Peeters-Scholte, M. Kriek, N. V. Knoers, D. Lindhout, B. P. C. Koeleman, M. J. A. van Kempen, E. H. Brilstra

**Affiliations:** 1 Department of Genetics, Center for Molecular Medicine, University Medical Center Utrecht, Lundlaan 6, 3584CG, Utrecht, The Netherlands; 2 Department of Genetics, University of Groningen, University Medical Center Groningen, Groningen, The Netherlands; 3 Department of Neurology, Erasmus MC, University Medical Center Rotterdam, Rotterdam, The Netherlands; 4 Department of Child Epileptology, Stichting Epilepsie Instellingen Nederland (S.E.I.N.), Heemstede, The Netherlands; 5 Adult and Child Neurology, Miller Children’s Hospital, Long Beach, CA USA; 6 Department of Paediatrics, Tjongerschans Hospital, Heerenveen, The Netherlands; 7 Department of Neurology, University of Groningen, University Medical Center Groningen, Groningen, The Netherlands; 8 Department of Clinical Genetics, Erasmus University Medical Center, Rotterdam, The Netherlands; 9 Division of Clinical Diagnostics, Ambry Genetics, Aliso Viejo, CA USA; 10 Department of Neurology, Leiden University Medical Center, Leiden, The Netherlands; 11 Department of Clinical Genetics, Leiden University Medical Center, Leiden, The Netherlands; 12 Department of Research, Stichting Epilepsie Instellingen Nederland (S.E.I.N), Zwolle, The Netherlands

**Keywords:** *PCDH19*, Mosaicism, Epilepsy, Intellectual disability

## Abstract

Pathogenic variants in the *PCDH19* gene are associated with epilepsy, intellectual disability (ID) and behavioural disturbances. Only heterozygous females and mosaic males are affected, likely due to a disease mechanism named cellular interference. Until now, only four affected mosaic male patients have been described in literature. Here, we report five additional male patients, of which four are older than the oldest patient reported so far. All reported patients were selected for genetic testing because of developmental delay and/or epilepsy. Custom-targeted next generation sequencing gene panels for epilepsy genes were used. Clinical data were collected from medical records. All patients were mosaic in blood for likely pathogenic variants in the *PCDH19* gene. In most, clinical features were very similar to the female phenotype, with normal development before seizure onset, which occurred between 5 and 10 months of age, clustering of seizures and sensitivity to fever. Four out of five patients had mild to severe ID and behavioural problems. We reaffirm the similarity between male and female *PCDH19*-related phenotypes, now also in a later phase of the disorder (ages 10–14 years).

**Electronic supplementary material:**

The online version of this article (doi:10.1007/s10048-017-0517-5) contains supplementary material, which is available to authorized users.

## Introduction

Pathogenic variants in *PCDH19* are associated with early onset, clustered epileptic seizures often provoked by fever, intellectual disability (ID) that can be present in variable degrees and behavioural disturbances such as autistic features, attention deficit, hyperactivity and aggression. The clinical features may resemble those of Dravet syndrome (phenotype MIM # 300088) [1–5].

*PCDH19* is located at Xq22.1 and codes for protocadherin-19, a transmembrane protein involved in neuronal organization and migration and cell-cell and cell-matrix adhesion [6–10]. It is highly expressed in the central nervous system [1]. *PCDH19*-related epilepsy shows a remarkable inheritance pattern: generally, females carrying heterozygous pathogenic variants are affected, whereas hemizygous male carriers are asymptomatic or only show psychiatric or behavioural symptoms [1, 2, 11, 12]. However, in 2009, the first affected male mosaic for a *PCDH19* pathogenic variant was described [2]. This finding gave rise to a theory of cellular interference as disease mechanism: disease occurs when two different cell populations exist (cells expressing the normal *PCDH19* protein and cells expressing a mutant form of the protein), as is true for heterozygous and mosaic pathogenic variants, but not for hemizygous pathogenic variants in males. These non-homogeneous cell populations are likely to disrupt cell-cell interactions, leading to disease [2].

Only four affected mosaic male patients have been described in literature until now, the oldest being 7 years old [2, 13, 14]. Here, we report five additional male patients with mosaic *PCDH19* mutations, of ages 2, 10, 13, 13 and 14 years old. We compared the male and female phenotypes.

## Methods

### Patients and molecular analysis

All five patients were selected for diagnostic genetic testing because of developmental delay and/or epilepsy. Genetic testing on DNA from lymphocytes was performed using a custom targeted next generation sequencing (NGS) gene panel for epilepsy genes (see online resource 1 for details). Mosaicism of *PCDH19* variants was determined based on the simultaneous presence of a variant allele and the reference allele, as *PCDH19* is located on the X-chromosome and all patients were male. The percentage of mosaicism was based on the percentage of reads that showed the alternate allele. *PCDH19* mosaic male patients from different centres were collected through personal communication between authors, meaning no structured cohort was tested. Detailed clinical data were collected from medical records. The parents of all patients gave informed consent for the publication of clinical data.

### Literature search

A literature study was carried out in the PubMed database to identify previously described patients with *PCDH19* pathogenic variants.

## Results

### Molecular analysis

All five patients carried a *PCDH19* variant in various degrees of mosaicism (patient A: c.1864G > C, p.Gly622Arg, 60%; patient B: c.840C > G, p.Tyr280*, 22%; patient C: c.462C > G, p.Tyr154*, 65%; patient D: c.1682C > G, p.Pro561Arg, 78%; patient E: c.799G > T p.Glu267*, 20%, RefSeq NM_001184880). According to American College of Medical Genetics and Genomics (ACMG) criteria, the variant of patient A was classified as likely pathogenic, and the variants of patient B-D were classified as pathogenic. The variants of patient B, C and E lead to a premature stopcodon; the variants of patient A and D were both predicted probably damaging and damaging by PolyPhen and SIFT, respectively. None of the variants was present in control databases (Database of Single Nucleotide Polymorphisms (dbSNP), NHLBI Exome Sequencing Project (ESP) and the the Exome Aggregation Consortium (ExAC) database). The Pro561Arg variant has been previously described in two affected female siblings with ID, microcephaly and seizures and was paternally transmitted [15]; the other variants are novel. All variants were confirmed de novo, except for the variant in patient D, whose parents were not tested. No other variants that could explain the phenotypes were found.

Clinical characteristics of the newly reported and previously described male patients with de novo *PCDH19* pathogenic variants are summarized and compared to those of previously published females in Table 1. See online resource for extensive clinical descriptions (online resource 2). Overall, all patients had normal development before seizure onset, occurring between 5 and 10 months of age, except for patient E. This patient had a delay in speech development, like his father, before onset of seizures and seizure onset occurred later, at 31 months. First seizure types were generalized clonic or clusters of focal or complex partial seizures. Later seizures were mainly complex partial seizures and primary or secondary generalized clonic and tonic-clonic seizures. In all five patients, seizures tended to cluster and could be provoked by fever. Four out of five patients had mild to severe ID, autism and additional behavioural problems.

**Table 1:** Clinical description of male and female patients carrying *PCDH19* likely pathogenic variants

Patient	A	B	C	D	E	F [2]	G [13]	H [14]	I [14]	Female patients^d^
Age at inclusion (years)	10	13	14	24 months	13	7	6	4	3,5	1–54
Variant^a^	c.1864G > C, p.Gly622Arg	c.840C > G, p.Tyr280*	c.462C > G, p.Tyr154*	c.1682C > G, p.Pro561Arg	c.799G > T, p.Glu267*	del *PCDH19*	c.605C > A, p.Ser202*	c.918C > G, p.Tyr306*	c.1352 C > T, p.Pro451Leu	*PCHD19* deletions, duplications, missense and nonsense variants
% of mosaicism	60% (blood)	22% (blood)	65% (blood)	78% (blood)	20% (blood)	100% (lymphocytes), 47% (fibroblasts)	50% (blood), 70% (buccal cells), 100% (urine sediment)	10% (lymphocytes, saliva, hair)	90% (lymphocytes, urine)	−
Technique used (number of alternate alleles/total read depth at base position)	NGS gene panel (92/153 reads)	NGS gene panel (77/380 reads)	NGS gene panel (38/59 reads)	NGS gene panel (351/450 reads)	NGS gene panel (19/93 reads)	Detection by microarray, estimation of % mosaicism by FISH (100 cells)	Detection by exome sequencing, estimation of % mosaicism by Sanger	NGS gene panel (157 reads)	NGS gene panel (135 reads)	Various
Sex	Male	Male	Male	Male	Male	Male	Male	Male	Male	Female
Exam at birth	Meconium stained amniotic fluid, bradycardia	Normal	Normal	Umbilical cord around neck, quick recovery	Normal	?	Normal	?	?	Normal
Development prior to sz onset	Normal	Normal	Normal	Normal	Speech delay	Normal	Normal	Normal	Normal	Normal
Sz onset age (months)	5	10	10	7	31	12	9	9	10	3–70 (most <12)
Sz type at onset	Generalized, clonic (fs)	Generalized clonic	Cluster of CP seizures	Clusters of focal sz, generalizing to GTC and tonic seizures	Clusters of focal sz, generalizing to one hemisphere	GTC (fs, prolonged, repetitive)	Focal myoclonic, tonic–clonic, FSsG	Afebrile hypotonic seizure with hypopnea (40 min)	24 h cluster of febrile sz with fixed gaze, loss of contact, upper limb hypertonia, and jerks (30–40 s.)	Febrile, febrile SE, afebrile; GTC, generalized tonic; hemiconvulsion; focal, FSsG; CP
Later sz types	CP, secondary generalized. Focal with affective symptoms, secondary generalized. Primairy generalized	Generalized clonic	CP, secondary tonic clonic, tonic, status	Focal, febrile, GTC, tonic, CP	CP, febrile	Hemiclonic, GTC, myoclonic jerks	Focal myoclonic, tonic–clonic, rapid secondary generalization	FS, focal tonic-vibratory	FS and afebrile seizure clusters, tonic. Often fearful screaming at start	FS or afebrile; generalized clonic, tonic, tonic-clonic or atonic; hemiclonic; focal, CP, FSsG, myoclonic; absences; SE
Clusters of sz	+ (fever related)	+	+	+	+	+	?	+	+	+
Focal sz with affective symptoms	+	−	+	−	+	?	?	?	+	+
Fever sensitivity	+	+	+	+	+	+	?	+	+	+
AEDs used and response^b^	VPA: -, LTG: -, CBZ:-	VPA: +	VPA:?, LEV: +/−, LZP (during clusters): -, TPM, CBZ, OXC, PHT, PHB, LTG, CLB, VGB:?	LEV:+/−, TPM: -, OXC:-, VPA:+, PHB:+, LSM:-, Diazepam:-	VPA:+; CBZ: +/−; OXC: +/−	VPA, CLB, CLN, TPM, STP:?	LEV: +/−, ZNS: +, multiple AEDs: -	VPA:?	PHB:?, VPA:	LEV, VPA, CLB, CLN, TPM, STP, LTG, PHB, CBZ, OXC, ZNS, NTZ, VGB, KBR, LZP: different responses
Current AEDs^b^	LEV, OXC, CLB, TPM	VPA	LEV	OXC, VPA, PHB, LEV	OXC	?	ZNS	−	PHB, VPA	Many different AED’s, some cases no AED’s
Sz outcome	1 cluster of sz per year (5–10 sz/cluster)	Last sz at age 10 years	Ongoing sz	Ongoing sz	Ongoing sz during febrile illness	Persistence of febrile sz in spite of treatment	Seizure free for 20 months	Seizure free age 14–42 months (no AEDs), since then 1 cluster of sz	4–5 clusters per year	Often seizures less frequent or seizure free at certain age (4–36 years)
EEG at onset	7–8 months: normal. 10–11 months: asymmetrical background activity, non-specific high voltage delta-activity occipital right > left.	Frequent generalized epileptic discharges	2 years: normal	Multiple focal discharges, right centroparietal, secondary generalization. Mild diffuse background slowing	Focal epileptic discharges left parietotemporal with generalization to one hemisphere (ictal)	?	Slower rhythm for age, mild diffuse disturbance. Infrequent right frontal, and rare left temporal sharp wave discharges, suggestive of epileptiform activity	Rare right frontotemporal sharp waves	Normal	Mostly normal
EEG at follow-up	3,5 years: normal	−	3 years: No epileptic activity, generalized and focal slow activity	-		?	?	?	Bilateral centroparietal onset of seizures	Normal, interictal spikes, generalized poly-spike waves, slow waves, slow background
Last EEG	6 years: normal background activity, diffuse fast activity mainly frontal. No epileptic activity.	−	4 years: No epileptic activity, generalized and focal slow activity	-	32 months: interictal EEG normal	?	?	?	Normal interictal	−
ID^c^	++ (estimated: slowed PMD, special education)	+ (IQ 66 at 5 years)	++ to +++	- (only delayed speech development, but no true ID yet, based on clinical evaluation)	+ (IQ 55 at 9 years)	++ to +++	+/− (IQ = 76)	+/− (GDQ 78 at 46 months, 72 at 52 months)	- (GDQ 101 at 30 months and 103 at 40 months)	- to +++
Developmental stagnation/regression	−	−	−	−	−	?	?	−	−	Regression in some cases
Language (words/sentences)	Delayed: words, sentences	Not delayed. First words at 12 months. Two-word sentences at 16 months	Sentences, stereotyped phrases	Mildly delayed speech development	Mildly delayed speech development	delay: words-sentences	?	?	?	Sometimes normal, often delayed, words-sentences, in rare cases absent
Behavioural/psychiatric disturbances	Autism, aggression, behavioural problems, ADHD	Autism, mood disorder	Autism spectrum disorder, anxiety	−	Behaviour problems resembling autism spectrum disorder, short attention span	Behavioural problems, autistic features	Irritability, aggression, rigidity, poor sleep, ADHD, anxiety, OCD, ODD	Compulsive and stereotyped behaviours	−	Prominent behavioural problems in most cases (autism, attention deficit, hyperactivity, aggression, emotional lability, impulsiveness, anxiety, jealousy, obsession, depression psychogenic nonepileptic sz)
Neurological examination	Crouched gait	Hypotonia	Motor delay and balance problems	Balance problems (medication induced), improving	Reduced coordination	Motor delay, ataxia	?	?	?	Mostly normal; hypotonia, dyspraxia, ataxia or motor delay in some
MRI images (age)	Expanded perivascular spaces (8 years)	Normal (7 years)	Widened peripheral subarachnoid spaces	Normal (7 months)	Normal (2,5 years)	?	Normal	? (CT: normal)	Normal (10 months)	Usually normal (mild atrophy/cortical dysplasia is rarely reported)
Additional comments	−	Pes plano valgus, obesity (BMI 23.3; +2.3 SD)	−	Hand food mouth disease			dysmorphic features: plagiocephaly occipital/parietal area, cupped ears, intradigital webbing of phalanges. Severe myopia			

*SZ* seizure(s), *AED* anti-epileptic drug, *PMD* psychomotor development, *GTC* generalized tonic-clonic, *CP* partial complex, *FS* fever sensitive, *SE* status epilepticus, *FSsG* focal seizure with secondary generalization, *CBZ* carbamazepine, *CLB* clobazam, *CLN* clonazepam, *KBR* potassium bromide, *LEV* levetiracetam, *LSM* lacosamide, *LTG* lamotrigine, *LZP* Lorazepam, *NTZ* nitrazepam, *OXC* oxcarbazepine, *PHB* phenobarbital, *PHT* phentytoin, *STP* stiripentol, *TPM* topiramate, *VGB* vigabatrin, *VPA* valproic acid, *ZNS* zonisamide, *GDQ* Griffiths Developmental Quotient

^a^RefSeq NM_001184880

^b^“−” not effective, “+/−” slight effect, “+” good effect

^c^“-” absent, “+/−” borderline, “+” mild, “++” moderate, “+++” severe

^d^[2–5, 11, 12, 15–29]

## Discussion

We here report five male patients with mosaic *PCDH19* likely pathogenic variants, which raises the total number of described male patients to nine [2, 13, 14]. Four of the currently described male patients are the oldest reported so far (ages 10, 13 twice and 14 years old), which gives the opportunity to investigate whether *PCDH19*-related phenotypes evolve the same way in male and female patients. Our current findings confirm previously reported observations of similar clinical features in male and female patients, also for older children [2, 13, 14]. Focal seizures with affective symptoms (fearful screaming) are very common in female patients and become more prevalent with an increasing age [26]. This distinctive seizure type is also reported in three male patients [14, current study]. These similarities suggest that there are no differences in clinical consequences between phenotypes caused by postzygotic *PCDH19* pathogenic variants in mosaic males, and phenotypes caused by heterozygous *PCDH19* pathogenic variants in females. This lends further support to the hypothesis that cellular interference is the main disease mechanism, as proposed by Depienne et al. [2] The increasing number of affected mosaic male patients undermines the theory of a compensating effect by the nonparalogous *PCDH11Y* gene in male patients, as proposed by Dibbens et al. [1]. It is highly unlikely that this gene would only compensate for a complete absence of *PCDH19* in hemizygous affected males, but not for a partial loss of *PCDH19* in mosaic males.

In female patients, seizure frequency often diminishes around puberty [3, 5, 11, 28–30], possibly due to hormonal changes [30]. Our 13-year old patient has been seizure free since the age of 10 years old; our 10-year-old patient only has one cluster of seizures per year. However, our other 13- and 14-year-old patients still have ongoing seizures. Although four of our male patients are the oldest reported until now, none of them has reached adolescence yet, and the numbers are still small, making it hard to draw definite conclusions. Nevertheless, since some of the few reported male patients already show a reduction in seizure frequency with increasing age, it seems unlikely that a declining seizure frequency is exclusively occurring in females and is related to female specific hormones.

We hypothesised that males with a mutated allele percentage around 50% in the brain, who would have an inherent high level of cellular interference, may show a more severe clinical picture than males with a lower or higher percentage of mosaicism. High or low percentages of mosaicism would resemble skewed X-inactivation in female patients, which has also been suggested to lead to a milder phenotype [5, 15], although no clear correlation has been shown [25, 29]. Indeed, in our cohort, the three patients with the lowest and highest percentages of mosaicism in blood are the least severely affected (patients B, D and E), and two previously described patients, one with borderline ID and one with normal intelligence showed percentages of mosaicism of 10 and 90%, respectively (patients H and I). However, patient G shows an equal number of mutated and wild-type alleles in blood and is also mildly affected, and patient F shows no mosaicism in blood cells at all, only in fibroblasts, but has moderate to severe ID and ongoing seizures. It is thus not possible to predict the phenotype based on the percentage of mosaicism in blood, most likely because it does not necessarily equal the percentage of mosaicism in the brain.

The number of identified male patients mosaic for *PCDH19* pathogenic variants increases [2, 13, 14], probably due to our improving abilities to detect mosaicism in general by using NGS techniques. It is now clear that *PCDH19* pathogenic variants can cause epilepsy both in males and females and that mosaicism for *PCDH19* pathogenic variants in males might be more common than previously thought. Because our five described patients were gathered through personal communication between authors from different diagnostic centres, no structured cohort with clearly defined inclusion criteria was tested, which makes it difficult to estimate the frequency of mosaic pathogenic *PCDH19* variants in male patients. Since the *PCDH19* and Dravet syndrome phenotypes show many similarities, testing a cohort of *SCN1A*-negative, male Dravet syndrome patients for mosaic *PCDH19* pathogenic variants could give more insight in the true incidence. In this cohort and in males with clinical features characteristic of a *PCDH19*-related disorder, NGS techniques with high coverage should be used to look for *PCDH19* pathogenic variants, as traditional Sanger Sequencing is not sensitive enough to reliably detect mosaicism. This overview helps create more knowledge about the disease course in male patients, which is extremely relevant for counselling those affected and their families. Reporting on more (older) patients in the future is essential for establishing a good understanding of prognosis in male patients.

## Electronic supplementary material

ESM 1 (PDF 94 kb)
