# Supplementary material for: Male patients affected by mosaic PCDH19 mutations: five new cases
Source: Neurogenetics. 2017 Jul 1;18(3):147–53. doi: 10.1007/s10048-017-0517-5 (PMC5522515; doi:10.1007/s10048-017-0517-5)
Supplement: Supplementary file 1 — (PDF 94 kb) [file 10048_2017_517_MOESM1_ESM.pdf]

## Supplemental data

### Online resource 1. Molecular analyses

Patient A, C and E were tested at University Medical Center Utrecht, the Netherlands, using Agilent Sure Select Enrichment. Base positions were genotyped using an 'in house' developed variant calling pipeline for NGS sequencing data. At an informative coverage of >15X, the genotyping sensitivity and specificity is > 99% (FN and FP << 1%, NGS versus SNP-array genotyped control DNA). The required horizontal coverage of target sequence (coding region including 20 bp flanking intron sequence) is > 95% at 15X. Patient B was tested at University Medical Center Groningen, the Netherlands. Base positions were genotyped using an 'in house' developed variant calling pipeline for NGS sequencing data. At an informative coverage of >20X, the genotyping sensitivity and specificity is > 99% (FN and FP << 1%, NGS versus genotyped control DNA). The required horizontal coverage of target sequence (coding region including 20 bp flanking intron sequence) is > 98% at 20X. Patient D was tested at Ambry Genetics, Aliso Viejo, USA. Sequence enrichment of the targeted coding exons and adjacent intronic nucleotides was carried out by a bait-capture methodology using long biotinylated oligonucleotide probes, and was followed by polymerase chain reaction (PCR), NGS sequencing with an analytical sensitivity of >99%.

All mutations were confirmed by conventional Sanger sequencing.

Genes enriched in the University Medical Center Utrecht epilepsy gene panel:
*ADSL, ALDH7A1, AMT, ARHGEF9, ARX, ASAH1, ATP1A2, ATP6AP2, ATRX, AUTS2, BRD2, CACNA1A, CACNA1H, CACNB4, CASK, CASR, CHD2, CDKL5, CHRNA2, CHRNA4, CHRNB2, CLN3, CLN5, CLN6, CLN8, CNKSR2, CNTNAP2, CPA6, CPT2, CTSD, CUL4B,*

*DCX, DEPDC5, DNAJC5, DYRK1A, EFHC1, EPM2A, FGD1, FOLR1, FOXG1, GABRA1, GABRB3, GABRD, GABRG2, GAMT, GCSH, GLDC, GLRA1, GLRB, GNAO1, GOSR2, GPC3, GPHN, GPR98, GRIA3, GRIN2A, GRIN2B, HCN1, HSD17B10, IQSEC2, KCNJ10, KCNMA1, KCNQ2, KCNQ3, KCNT1, KCTD7, KDM5C, LGI1, MAGI2, MAPK10, MBD5, ME2, MECP2, MED12, MEF2C, MFSD8, MTHFR, NHLRC1, NRXN1, OFD1, OPHN1, PAK3, PCDH19, PHF6, PIGA, PIGN, PIGT, PLCB1, PLP1, PNKP, PNPO, PPT1, PPT2, PQBP1, PRICKLE1, PRICKLE2, PRRT2, RAB39B, RANBP2, RNASEH2A, RNASEH2B, RNASEH2C, ROGD1, SAMHD1, SCARB2, SCN1A, SCN1B, SCN2A, SCN8A, SLC19A3, SLC25A22, SLC2A1, SLC6A5, SLC6A8, SLC9A1, SLC9A6, SMC1A, SMS, SPTAN1, SRPX2, ST3GAL3, STXBP1, SYN1, SYNGAP1, SYP, SZT2, TBC1D24, TBCE, TCF4, TPP1, TREX1, UBE3A, ZEB2*

Genes enriched in the University Medical Center Groningen epilepsy gene panel:

*CLCN2, GABRD, GABRG2, GPR98, PCDH19, RANBP2, SCN1A, SCN1B, SCN2A, SCN9A, SLC9A1, TBC1D24*

Genes enriched in the Ambry Genetics epilepsy gene panel:

*ALDH7A1, ARHGEF9, ARX, ATP13A2, ATP1A2, CACNA1A, CASK, CDKL5, CHD2, CHRNA2, CHRNA4, CHRNB2, CLN3, CLN5, CLN6, CLN8, CNTNAP2, CRH, CSTB, CTSD, CTSF, DCX, DEPDC5, DNAJC5, DNM1, DYNC1H1, DYRK1A, EEF1A2, EPM2A, FLNA, FOLR1, FOXG1, GABRA1, GABRB3, GABRG2, GAMT, GATM, GNAO1, GOSR2, GRIN1, GRIN2A, GRIN2B, GRN, HCN1, HNRNPU, IQSEQ2, KCNA2, KCNC1, KCNJ10, KCNQ2, KCNQ3, KCNT1, KCTD7, KIAA2022, LGI1, MECP2, MEF2C, MFSD8, NHLRC1, NRXN1, PDCH19, PIGA, PLCB1, PNKP, PNPO, POLG, PPT1, PRICKLE1, PRRT2, PURA, SCARB2, SCN1A, SCN1B, SCN8A, SIK1, SLC13A5, SLC25A22, SLC2A1, SLC35A2, SLC6A1, SLC9A6, SMC1A, SNAP25,*

*SPTAN1, ST3GAL3, STX1B, STXBP1, SYN1, SYNGAP1, SZT2, TBC1D24, TBL1XR1, TCF4, TPP1, TSC1, TSC2, UBE3A, WDR45, ZEB2.*

**Online resource 2. Clinical descriptions**

**Patient A**

Patient A is a ten year old boy, who had generalized clonic seizures starting at the age of five months. These seizures typically occurred in clusters of 20 to 30 seizures during one day every month. From age three years most clusters were triggered by infections with fever; before that there was no association with fever. He was initially treated with valproic acid, lamotrigine and carbamazepine, which was unsuccessful. Best epilepsy control was achieved with a combination of clobazam, topamirate, oxcarbazepine and levetiracetam, although he still has fever related clusters of seizures. His brain MRI showed enlarged perivascular spaces without other abnormalities. His first EEG at the age of 7 months was normal, whereas his second EEG at age 10 months showed an asymmetrical background pattern with nonspecific high voltage delta-activity in the occipital region, predominantly at the right side. He has a global developmental delay and attends a special school for children with learning and behavioural problems. He has been diagnosed with autism. At age nine years he developed a crouched gait.

**Patient B**

This 13-year-old boy had generalized clonic seizures since the age of 10 months. Seizures were triggered by fever, mostly due to middle ear infections and infections of the upper respiratory tract or urinary tract. Only once a seizure occurred in the absence of infection or fever. Seizures tended to cluster, with multiple seizures occurring during disease episodes. He has been treated with valproic acid, and had his last seizures at the age of 10 years. An EEG at the age of 10 months showed generalized epileptic discharges. Brain MRI at the age of 7 years was normal. He has a global developmental delay and a mild to moderate intellectual disability (at age 6 years his TIQ was 50, with a VIQ of 55 and a PIQ of 55 (SON-R)). He attends a special school for

children with learning problems. He has mood swings and was further diagnosed with a pervasive developmental disorder not otherwise specified (PDD-NOS). His mother had one febrile seizure during infancy; family history was otherwise negative for epilepsy. He has pes planovalgus and mild obesity, but no particular dysmorphic facial characteristics.

**Patient C**

Patient C is a 14-year-old boy who started having seizures at the age of 10 months. Clusters of complex partial and tonic-clonic, or tonic seizures occurred every 1 to 2 months, with seizure-free intervals of several months to one year. Clusters were provoked by fever and stress. They were refractory to antiepileptic drugs, leading to admittance to the intensive care unit at least nine times each year. The first interictal EEG was normal; later interictal EEGs showed generalized and focal slow activity, compatible with atypical diffuse encephalopathy. Developmental delay was 1.5 year at age three years, both on mental and motor scales. At age 5;11 years, the Bayley's Scales (BSID-II-NL) revealed a developmental level of 2;2 and 2;3 years on the mental and motor scales, respectively. At age 14 years old, he makes short, two to three-word sentences, spoken mostly in a high pitched tone or a whispering voice. Words and sentences may be repeated over and over again. He is unable to do basic school tasks like reading or writing. Behaviour is characterized by rigidity, fixations and perseverations, diagnosed as an autistic spectrum disorder. Anxiety is present as well. He attends a school for children with epilepsy and learning problems and has a moderate to severe intellectual disability.

**Patient D**

This 24 month old boy presented at age 7 months with a cluster of febrile focal and generalized tonic seizures which was stabilized with valproic acid. He was admitted to the hospital following

a second episode of seizure clusters precipitated by fever two months later. Three more admissions with clustered seizures and fever followed. Seizures always occur out of sleep. An EEG showed multiple focal discharges, right centroparietal, with secondary generalization and mild diffuse background slowing. Current medication consists of oxcarbazepine, levetiracetam, phenobarbital and valproate. Speech development is mildly delayed. There is no known family history of febrile seizures or epilepsy.

**Patient E**

This 13 year old boy was known with a delay in speech development, when, at the age of 2.5 years he had his first cluster of seizures with gazing, head turn to the left, blue lips and pale face, during a period of febrile illness. An ictal EEG was asymmetrical with background slowing at the left with epileptic discharges at the left parieto-temporal side with spreading to both hemispheres. He was treated with valproic acid for 6 months. MRI of the brain was normal. At the age of 4;7 years a developmental age of 2.6 years was measured (SON-R, 2.5-7 years). After discontinuation of valproic acid, seizures reoccurred during febrile illnesses and valproic acid was restarted until the age of 5.5 years, when it was discontinued because of behavioural problems. At age 9 years his IQ score was 55 (WISC), and he showed some autism characteristics but did not fulfil diagnostic criteria of autism spectrum disorder . At the age of 11 years he had multiple episodes with recurrent seizures during febrile illnesses and after BMR vaccination. He was treated with clobazam during  periods of fever, resulting in reduced seizure frequency and duration. At the age of 13 years oxcarbazepine treatment was started because of a further increase in seizure frequency with moderate effect. Currently he has a mild to moderate, mainly cognitive, developmental delay. He is not able to speak sentences fluently. His reading, writing and calculating skills are comparable to those of a 7-year old. There is no known family

history of febrile seizures or epilepsy, but his father had a stutter during childhood and experienced difficulties with learning how to read.
